# Real-world assessment of low-density lipoprotein cholesterol goal, disease burden and financial burden among patients with atherosclerotic cardiovascular disease in Singapore—A single-centre retrospective cohort study

**DOI:** 10.3389/fcvm.2026.1815535

**Published:** 2026-06-12

**Authors:** Aaron KH Ho, Aloysius Sheng-Ting Leow, Sarah Ming-Li Tan, Vincent Wei Jun Sim, Qian Yan, Ching-Hui Sia, Oliver Simon, Kian-Keong Poh

**Affiliations:** 1 Department of Medicine, National University Health System, Singapore; 2 Department of Cardiology, National University Heart Centre, Singapore; 3 Department of Medicine, Yong Loo Lin School of Medicine, National University of Singapore, Singapore; 4 Medical Affairs Department, Novartis (Singapore) Pte Ltd, Singapore

**Keywords:** acute coronary syndrome, atherosclerotic cardiovascular disease, lipid management, low-density lipoprotein cholesterol (LDL-C), real-world, Singapore

## Abstract

**Background:**

Despite strong evidence supporting intensive low-density lipoprotein cholesterol (LDL-C) lowering in high- and very-high-risk patients to reduce atherosclerotic cardiovascular disease (ASCVD), real-world attainment of recommended lipid targets remains poor, particularly in Asian populations. This study aims to evaluate LDL-C goal attainment, treatment patterns, and the clinical and economic burden among post-myocardial infarction patients in Singapore.

**Materials and methods:**

We conducted a retrospective, observational cohort study of 300 consecutive post-myocardial infarction patients admitted to the National University Heart Centre, Singapore, between January and June 2022, using electronic medical records to assess LDL-C goal attainment, disease burden, and direct healthcare costs. The primary outcome was attainment of prespecified LDL-C thresholds, including <2.6 mmol/L according to local Ministry of Health guidance and <1.4 mmol/L according to ESC/EAS very-high-risk recommendations. Attainment of <1.8 mmol/L was analysed as an additional intermediate threshold. Secondary outcomes included treatment patterns, major adverse cardiovascular events (MACE), healthcare utilisation, and costs. Comparisons were made using chi-square tests for categorical variables and independent samples *t*-tests or Mann–Whitney *U*-tests for continuous variables.

**Results:**

The mean age was 60.9 ± 12.2 years, 82.0% were male, and baseline LDL-C was 3.1 ± 1.3 mmol/L. At one year, 80.7% achieved <2.6 mmol/L, 47.3% achieved <1.8 mmol/L, and 18.3% achieved <1.4 mmol/L. High-intensity statin use increased from 23.0% at baseline to 95.7% at discharge, while ezetimibe and PCSK9 inhibitor use remained low at 13.0% and 0%, respectively. LDL-C control was not significantly associated with MACE at one year (*p* = 0.412). Estimated combined annualised direct costs were SGD 1,003 per patient, including statin therapy, ED attendance/inpatient admission, and outpatient care.

**Conclusion:**

These findings demonstrate that LDL-C target attainment in Singapore remains suboptimal, particularly among very high-risk ASCVD patients, despite widespread statin use. Clinically, this supports earlier protocol-driven combination lipid-lowering therapy rather than delayed statin-only escalation. Early ezetimibe initiation, timely lipid reassessment within 4–12 weeks, and prompt consideration of PCSK9 inhibitors or other novel agents for persistently above-target patients should be embedded into structured follow-up pathways. Broader access and subsidy for advanced therapies may help improve LDL-C target attainment and reduce residual cardiovascular risk in Singapore.

## Introduction

Cardiovascular disease (CVD) remains the leading cause of death globally, accounting for nearly 31% of all deaths (1). Over the last two decades, the global burden of CVD has increased substantially, with cases more than doubling from 311 million to 626 million and CVD deaths increasing from 13.1 million to 19.2 million from 1990 to 2023, alongside significant increase in disability-adjusted life years (DALYs) (2, 3). In Singapore, CVD accounted for 29.3% of all deaths and 14.2% of the nation's total DALYs, with estimated annual healthcare costs reaching USD 8.1 billion (4, 5).

Hyperlipidaemia, particularly elevated low-density lipoprotein cholesterol (LDL-C), is a well-established and modifiable risk factor for atherosclerotic cardiovascular disease (ASCVD). Robust evidence from randomised trials and meta-analyses has demonstrated that each 1 mmol/L reduction in LDL-C confers a proportional 20%–25% reduction in major vascular events, consistent across sexes, ages, and baseline risk categories (6, 7). Consequently, international guidelines advocate for intensive LDL-C lowering, particularly in high- and very high-risk patients. The 2018 American Heart Association (AHA)/American College of Cardiology (ACC) guidelines recommend a target LDL-C of <1.8 mmol/L for very high-risk patients (8), while the 2025 focused update of the 2019 European Society of Cardiology (ESC)/European Atherosclerotic Society (EAS) guidelines adopt a stricter goal of <1.4 mmol/L (9, 10).

High-intensity statins are the preferred first-line therapy, followed by combination treatment with ezetimibe and, if needed, proprotein convertase subtilisin/kexin type 9 (PCSK9) inhibitors. Further, newer lipid-lowering agents have expanded treatment options for patients unable to reach LDL-C goals with conventional therapy (8–10). Inclisiran, a small interfering RNA (siRNA) that inhibits hepatic PCSK9 synthesis, reduces LDL-C by up to 50%, with sustained lipid-lowering effects for at least 18 months. Following an initial dose and a second dose at 3 months, inclisiran is administered twice yearly thereafter (11). Bempedoic acid, an oral ATP citrate lyase inhibitor acting upstream of HMG-CoA reductase, lowers LDL-C by approximately 30% as monotherapy and up to 50% when combined with ezetimibe (12).

Despite these advancements, LDL-C target attainment remains suboptimal in Asia (13, 14). In Singapore, an observational study of mainly Asian patients with stable coronary heart disease (CHD) or acute coronary syndrome (ACS) found that 54.3% of CHD patients and 76.2% of ACS patients did not meet the national LDL-C goal (<1.8 mmol/L) at baseline. Patients receiving lipid-lowering therapies (LLTs) at baseline had significantly lower LDL-C levels than untreated patients, underscoring the importance of early and aggressive therapy (15). Nevertheless, real-world goal attainment, treatment patterns, and healthcare costs with regards to LDL-C among Singaporean patients with established ASCVD remain incompletely characterised. Most existing evidence comes from Western populations, and differences in healthcare access, treatment practices, subsidy structures, follow-up pathways, and ethnic risk profiles may limit direct extrapolation to Asian settings (16, 17).

Hence, the aim of this study was to evaluate LDL-C goal attainment, lipid-lowering treatment patterns, cardiovascular outcomes, healthcare utilisation, and direct medical costs in a real-world cohort of post-myocardial infarction patients in Singapore. We hypothesised that a substantial proportion of patients would not achieve guideline-recommended LDL-C targets, particularly the very-high-risk threshold of <1.4 mmol/L, reflecting underuse of combination lipid-lowering therapy and delayed lipid reassessment.

## Materials and methods

### Study design and population

This was a retrospective, observational cohort study conducted at the National University Heart Centre Singapore, a tertiary cardiac centre in Singapore. Consecutive post-myocardial infarction patients admitted between January and June 2022 were identified from electronic medical records and followed for 12 months ± 1 month after index admission. Ethics approval was obtained from the National Healthcare Group Domain Specific Review Board (DSRB reference number: 2022/00162). The requirement for informed consent was waived because of the retrospective design and use of de-identified clinical data.

**Figure 1:**
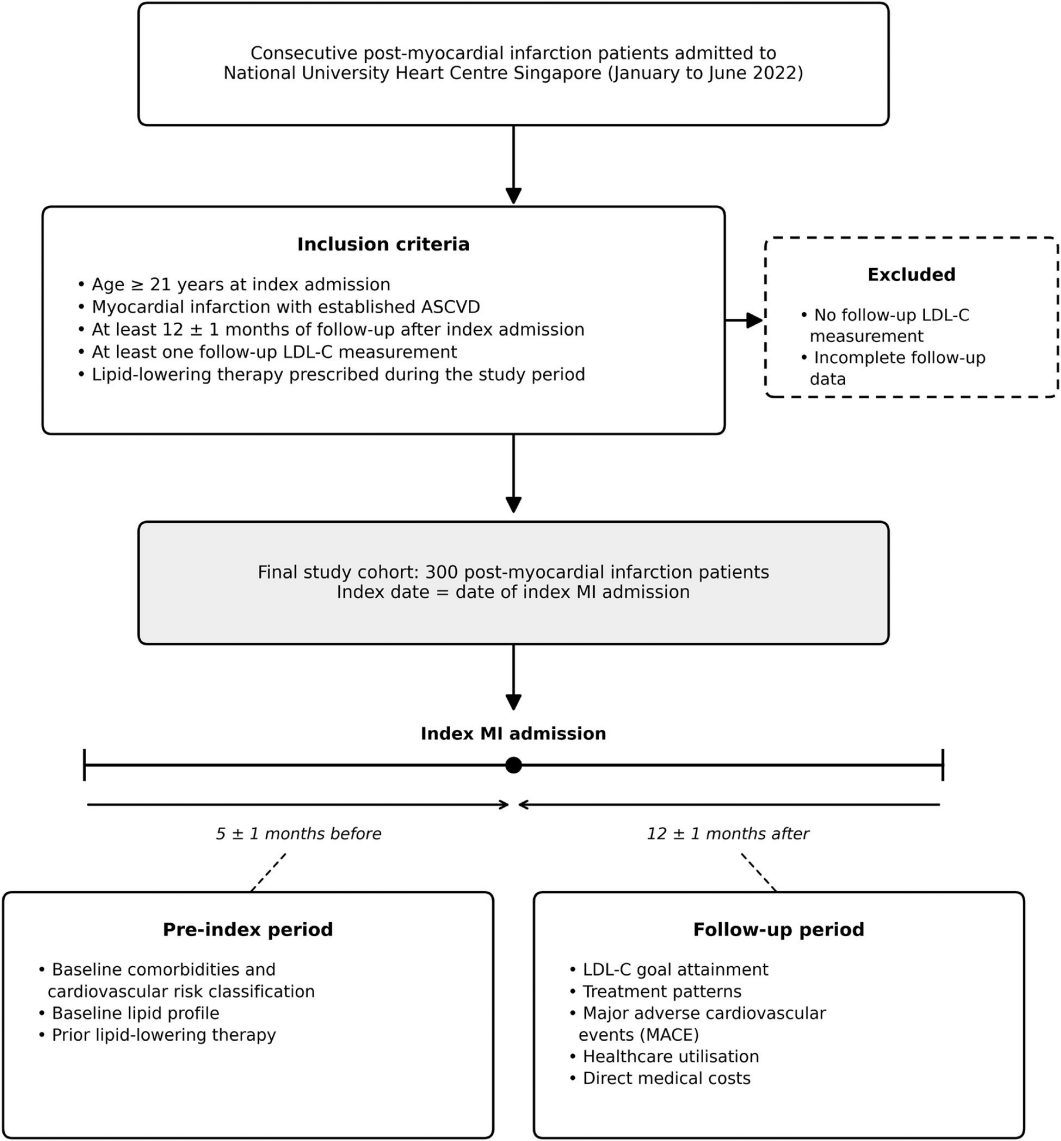
Study design.

Patients were included if they met all of the following criteria: age ≥21 years at the index admission; diagnosis of myocardial infarction with established ASCVD; availability of at least one year ± 1 month of follow-up data after the index admission; at least one recorded LDL-C measurement during follow-up; and prescription of lipid-lowering therapy during the study period. Patients were excluded if they did not meet these criteria, had no follow-up LDL-C measurement, or had incomplete follow-up data (Figure 1).

A pre-index period of 5 months ±1 month was used to capture relevant baseline history, including prior diagnoses, cardiovascular risk classification, baseline lipid profile, and prior LLTs. Follow-up extended for 12 months ±1 month after the index admission. Major adverse cardiovascular events (MACE) were adjudicated using predefined criteria, including the Fourth Universal Definition of Myocardial Infarction, and reviewed by at least two independent physicians (18).

### Sample size and power calculation

A sample size calculation was performed before analysis. Assuming an LDL-C <1.8 mmol/L goal attainment rate of 35% compared with a minimum clinically relevant rate of 26%, a one-sided binomial test with 90% power and a significance level of 0.05 required at least 230 patients (Supplementary Table S1). After accounting for an anticipated 10% rate of incomplete data, a minimum sample size of 256 patients was targeted. The final cohort included 300 patients.

### Measurements

Demographic, clinical, laboratory, medication, procedural, healthcare utilisation, and cost data were extracted from the electronic medical record. Variables collected included age, sex, ethnicity, cardiovascular risk factors, comorbidities, disease presentation, anthropometric measurements, vital signs, clinical examination findings, baseline and follow-up laboratory investigations, revascularisation status, discharge medications, healthcare utilisation, direct medical costs, and clinical outcomes. Lipid-lowering therapy was classified according to statin intensity and use of non-statin agents, including ezetimibe, fibrates, PCSK9 inhibitors, inclisiran, and other lipid-lowering medications.

Lipid profile measurements, including total cholesterol, HDL-C, triglycerides, and LDL-C, were performed by the institutional clinical laboratory using routine standardised protocols on the Abbott Alinity platform (Abbott Laboratories, Abbott Park, IL, USA). LDL-C was calculated using the Friedewald equation. Left ventricular ejection fraction was obtained from clinically indicated transthoracic echocardiography reports performed as part of routine care. As imaging parameters were extracted from final clinical reports rather than re-analysed for research purposes, intra-observer and inter-observer variability were not assessed.

### Study endpoints

The primary endpoint was the proportion of ASCVD patients achieving prespecified LDL-C treatment goals within the first year ±1 month after the index admission, defined as <2.6 mmol/L per the 2016 Singapore Ministry of Health Clinical Practice Guidelines on Lipids (19) and <1.4 mmol/L per ESC/EAS guidelines (10). These local guidelines have since been updated in 2023, lowering the LDL-C goals for high- and very-high-risk patients to align more closely with international recommendations. Attainment of <1.8 mmol/L was also analysed as an intermediate threshold to allow comparison with earlier guideline targets and prior real-world studies. Secondary outcomes included the proportion of patients stratified into LDL-C categories of <1.4 mmol/L, ≥1.4 to <1.8 mmol/L, and ≥1.8 to <2.6 mmol/L; treatment patterns with statins (low-, moderate-, high-intensity) and other LLTs, alone or in combination, based on the definitions from the 2018 AHA/ACC guidelines (Table 1) (8); disease burden including new-onset ASCVD events, subsequent events of the same or different type, and coronary revascularisation; healthcare resource utilisation (HCRU); and the direct costs of ASCVD management, including hospitalisations, emergency department visits, outpatient specialist care, and medication costs.

**Table 1:** Definitions of low-, moderate-, and high- statin regimens.

Low intensity	Moderate intensity	High intensity
Simvastatin 10 mg Pravastatin 10–20 mg Lovastatin 20 mg Fluvastatin 20–40 mg	Atorvastatin 10–20 mg Rosuvastatin 5–10 mg Simvastatin 20–40 mg Pravastatin 40–80 mg Lovastatin 40–80 mg Fluvastatin XL 80 mg Fluvastatin 40 mg bid Pitavastatin 1–4 mg	Atorvastatin 40–80 mg Rosuvastatin 20–40 mg

### Statistical analysis

Continuous variables were assessed for normality using the Shapiro–Wilk test and visual inspection of histograms and Q–Q plots. Normally distributed continuous variables were reported as mean ± standard deviation and compared using independent samples *t*-tests. Non-normally distributed variables were reported as median with interquartile range and compared using the Mann–Whitney *U*-test. Categorical variables were reported as frequencies and percentages and compared using chi-square tests or Fisher's exact tests, as appropriate.

The cost of statins was estimated from the latest available pharmacy list, while the costs of emergency department attendance, hospitalisation in subsidised C-class general wards, and outpatient clinic visits were based on published fee benchmarks. Statistical significance was set at *p* < 0.05. All analyses were performed using R Statistical Software version 4.4.1 and RStudio version 2024.9.0. Missing data were minimal and did not significantly affect the results.

### Subgroup analysis

No formal subgroup analyses were prespecified. Additional subgroup analyses by sex, ethnicity, diabetes status, or other clinical strata were not planned because the study was designed primarily to estimate LDL-C goal attainment and treatment patterns, rather than to test subgroup-specific associations with clinical outcomes.

## Results

A total of 300 consecutive post-myocardial infarction patients were included. The mean age was 60.9 ± 12.2 years, with 82.0% (*n* = 246) male and 53.3% (*n* = 160) Chinese. Cardiovascular risk factors were highly prevalent: hyperlipidaemia in 70.0% (*n* = 210), hypertension in 59.3% (*n* = 178), diabetes mellitus in 43.0% (*n* = 129), and current or former smoking in 46.7% (*n* = 140). A family history of ischaemic heart disease was present in 4.7% (*n* = 14), and familial hypercholesterolaemia in 1.3% (*n* = 4). The mean baseline LDL-C was 3.1 ± 1.3 mmol/L (Tables 2–4).

**Table 2:** Baseline characteristics, treatment patterns, and outcomes stratified by LDL-C target attainment at <2.6 mmol/L.

Variables	Overall *N* = 300	Did not reach target *N* = 58	LDL target < 2.6 *N* = 242	*p*-value
Age (years), mean (SD)	60.9 (12.2)	57.1 (11.1)	61.8 (12.3)	0.009
Female, *n* (%)	54 (18.0)	8 (13.8)	46 (19.0)	0.353
Race, *n* (%)				0.014
Chinese	160 (53.3)	24 (41.4)	136 (56.2)	
Malay	58 (19.3)	19 (32.8)	39 (16.1)	
Indian	72 (24.0)	15 (25.9)	57 (23.6)	
Others	10 (3.3)	0 (0.0)	10 (4.1)	
Family history, *n* (%)				
IHD	14 (4.7)	1 (1.7)	13 (5.4)	0.319
Stroke	3 (1.0)	0 (0.0)	3 (1.2)	>0.999
Co-morbidities, *n* (%)				
Previous or current smoking	140 (46.7)	36 (62.1)	104 (43.0)	0.009
DM	129 (43.0)	25 (43.1)	104 (43.0)	0.986
HLD	210 (70.0)	46 (79.3)	164 (67.8)	0.085
FH	4 (1.3)	1 (1.7)	3 (1.2)	0.579
HTN	178 (59.3)	37 (63.8)	141 (58.3)	0.441
Prothrombotic state	16 (5.3)	5 (8.6)	11 (4.5)	0.206
AF	26 (8.7)	4 (6.9)	22 (9.1)	0.594
Known IHD (non-AMI)	24 (8.0)	7 (12.1)	17 (7.0)	0.277
Previous CIED implantation	4 (1.3)	0 (0.0)	4 (1.7)	>0.999
Known HF	34 (11.3)	7 (12.1)	27 (11.2)	0.844
Previous stroke/TIA	28 (9.3)	7 (12.1)	21 (8.7)	0.425
PAD	15 (5.0)	2 (3.4)	13 (5.4)	0.744
Anxiety	1 (0.3)	1 (1.7)	0 (0.0)	0.194
Depression	4 (1.3)	2 (3.4)	2 (0.8)	0.170
MCI/dementia	7 (2.3)	2 (3.4)	5 (2.1)	0.624
Chronic liver disease	14 (4.7)	4 (6.9)	10 (4.1)	0.485
Chronic kidney disease	34 (11.3)	6 (10.3)	28 (11.6)	0.791
Past COVID-19	11 (3.7)	2 (3.4)	9 (3.7)	>0.999
HIV	1 (0.3)	1 (1.7)	0 (0.0)	0.193
Labs at baseline, mean (SD)				
TG	1.8 (1.1)	1.9 (1.1)	1.7 (1.1)	0.298
TC	4.9 (1.4)	5.8 (1.4)	4.7 (1.4)	<0.001
LDL	3.1 (1.3)	3.9 (1.2)	2.9 (1.2)	<0.001
HDL	1.1 (0.3)	1.1 (0.3)	1.1 (0.3)	0.922
HbA1c	6.8 (1.8)	7.3 (2.0)	6.7 (1.7)	0.024
FPG	7.8 (3.7)	8.6 (4.3)	7.6 (3.5)	0.085
Medications at baseline, *n* (%)				
Aspirin	77 (25.7)	17 (29.3)	60 (24.8)	0.479
Any p2y12 inhibitors	29 (9.7)	3 (5.2)	26 (10.7)	0.197
OAC	6 (2.0)	0 (0.0)	6 (2.5)	0.600
BB	77 (25.7)	11 (19.0)	66 (27.3)	0.193
CCB	69 (23.0)	11 (19.0)	58 (24.0)	0.416
Nitrate	34 (11.3)	4 (6.9)	30 (12.4)	0.235
Ranolazine	0 (0.0)	0 (0.0)	0 (0.0)	>0.999
Trimetazidine	4 (1.3)	1 (1.7)	3 (1.2)	0.579
ACEi/ARB/ARNI	88 (29.3)	15 (25.9)	73 (30.2)	0.518
MRA	10 (3.3)	0 (0.0)	10 (4.1)	0.218
Any statins	140 (46.7)	25 (43.1)	115 (47.5)	0.545
Ezetimibe	25 (8.3)	3 (5.2)	22 (9.1)	0.434
Fibrates	10 (3.3)	2 (3.4)	8 (3.3)	>0.999
PCSK9i	1 (0.3)	0 (0.0)	1 (0.4)	>0.999
Inclisiran	0 (0.0)	0 (0.0)	0 (0.0)	N/A
Other lipid lowering medications	0 (0.0)	0 (0.0)	0 (0.0)	N/A
All lipid lowering medications	145 (48.3)	25 (43.1)	120 (49.6)	0.375
Combination lipid lowering medications	29 (9.7)	5 (8.6)	24 (9.9)	0.764
SGLT2i	26 (8.7)	5 (8.6)	21 (8.7)	0.989
Other OHGAs	88 (29.3)	11 (19.0)	77 (31.8)	0.053
Insulin	24 (8.0)	2 (3.4)	22 (9.1)	0.187
Statin intensity at baseline, *n* (%)				0.678
Low intensity	14 (4.7)	2 (3.4)	12 (5.0)	
Moderate intensity	57 (19.0)	8 (13.8)	49 (20.2)	
High intensity	69 (23.0)	15 (25.9)	54 (22.3)	
Medications on discharge, *n* (%)				
Aspirin	287 (95.7)	58 (100.0)	229 (94.6)	0.080
Any p2y12 inhibitors	289 (96.3)	55 (94.8)	234 (96.7)	0.450
OAC	38 (12.7)	9 (15.5)	29 (12.0)	0.467
BB	253 (84.3)	52 (89.7)	201 (83.1)	0.214
CCB	32 (10.7)	9 (15.5)	23 (9.5)	0.183
Nitrate	157 (52.3)	25 (43.1)	132 (54.5)	0.117
Ranolazine	1 (0.3)	0 (0.0)	1 (0.4)	>0.999
Trimetazidine	6 (2.0)	1 (1.7)	5 (2.1)	>0.999
ACEi/ARB/ARNI	220 (73.3)	42 (72.4)	178 (73.6)	0.860
MRA	23 (7.7)	5 (8.6)	18 (7.4)	0.784
Any statins	295 (98.3)	56 (96.6)	239 (98.8)	0.248
Ezetimibe	39 (13.0)	10 (17.2)	29 (12.0)	0.285
Fibrates	9 (3.0)	1 (1.7)	8 (3.3)	>0.999
PCSK9i	0 (0.0)	0 (0.0)	0 (0.0)	N/A
Inclisiran	0 (0.0)	0 (0.0)	0 (0.0)	N/A
Other lipid lowering medications	1 (0.3)	0 (0.0)	1 (0.4)	>0.999
All lipid lowering medications	297 (99.0)	56 (96.6)	241 (99.6)	0.097
Combination lipid lowering medications	46 (15.3)	10 (17.2)	36 (14.9)	0.653
SGLT2i	35 (11.7)	6 (10.3)	29 (12.0)	0.727
Other OHGAs	115 (38.3)	22 (37.9)	93 (38.4)	0.944
Insulin	32 (10.7)	3 (5.2)	29 (12.0)	0.131
Statin intensity on discharge, *n* (%)				0.161
Low intensity	1 (0.3)	0 (0.0)	1 (0.4)	
Moderate intensity	7 (2.3)	3 (5.2)	4 (1.7)	
High intensity	287 (95.7)	53 (91.4)	234 (96.7)	
LVEF (%), mean (SD)	47.8 (11.8)	46.2 (10.7)	48.2 (12.1)	0.250
PCI prior to discharge, *n* (%)	235 (78.3)	43 (74.1)	192 (79.3)	0.388
CABG prior to discharge, *n* (%)	12 (4.0)	2 (3.4)	10 (4.1)	>0.999
Repeat LDL	2.0 (0.9)	3.5 (0.9)	1.7 (0.4)	<0.001
Duration to repeat LDL (months), mean (SD)	9.5 (4.2)	10.4 (4.8)	9.2 (4.0)	0.045
Duration of follow-up (years), mean (SD)	1.0 (0.3)	0.9 (0.3)	1.0 (0.3)	0.661
Outcomes, *n* (%)				
All-cause mortality	12 (4.0)	2 (3.4)	10 (4.1)	>0.999
CV mortality	10 (3.3)	2 (3.4)	8 (3.3)	>0.999
ACS	29 (9.7)	5 (8.6)	24 (9.9)	0.764
Stroke/TIA	7 (2.3)	1 (1.7)	6 (2.5)	>0.999
PAD	3 (1.0)	0 (0.0)	3 (1.2)	>0.999
HF hospitalisation	32 (10.7)	8 (13.8)	24 (9.9)	0.390
MACE^a^	39 (13.0)	6 (10.3)	33 (13.6)	0.503
Number of readmissions per patient, mean (SD)	1.1 (0.4)	1.1 (0.3)	1.2 (0.5)	0.165
Average length of stay (days), mean (SD)	4.7 (5.2)	5.8 (8.5)	4.4 (3.8)	0.205
Number of outpatient clinic visits per patient, mean (SD)	3.2 (1.6)	3.1 (1.9)	3.2 (1.6)	0.950
Annualised cost ($), mean (SD)				
Statin medication	677 (1,108)	785 (1,577)	651 (965)	0.414
ED attendance + inpatient admission	144 (354)	220 (619)	126 (251)	0.072
Outpatient clinic visit	182 (170)	212 (303)	175 (117)	0.137
Subtotal medical service	1,003 (1,235)	1,215 (1,763)	952 (1,070)	0.146

a MACE included outcomes of CV mortality, ACS, stroke/TIA.

**Table 3:** Baseline characteristics, treatment patterns, and outcomes stratified by LDL-C target attainment at <1.8 mmol/L.

Variables	Overall *N* = 300	Did not reach target *N* = 158	LDL target <1.8 *N* = 142	*p*-value
Age (years), mean (SD)	60.9 (12.2)	59.8 (12.1)	62.1 (12.4)	0.095
Female, *n* (%)	54 (18.0)	30 (19.0)	24 (16.9)	0.639
Race, *n* (%)				0.081
Chinese	160 (53.3)	76 (48.1)	84 (59.2)	
Malay	58 (19.3)	36 (22.8)	22 (15.5)	
Indian	72 (24.0)	38 (24.1)	34 (23.9)	
Others	10 (3.3)	8 (5.1)	2 (1.4)	
Family history, *n* (%)				
IHD	14 (4.7)	9 (5.7)	5 (3.5)	0.373
Stroke	3 (1.0)	3 (1.9)	0 (0.0)	0.250
Co-morbidities, *n* (%)				
Previous or current smoking	140 (46.7)	82 (51.9)	58 (40.8)	0.055
DM	129 (43.0)	61 (38.6)	68 (47.9)	0.105
HLD	210 (70.0)	113 (71.5)	97 (68.3)	0.545
FH	4 (1.3)	2 (1.3)	2 (1.4)	>0.999
HTN	178 (59.3)	92 (58.2)	86 (60.6)	0.681
Prothrombotic state	16 (5.3)	9 (5.7)	7 (4.9)	0.768
AF	26 (8.7)	14 (8.9)	12 (8.5)	0.900
Known IHD (non-AMI)	24 (8.0)	11 (7.0)	13 (9.2)	0.485
Previous CIED implantation	4 (1.3)	2 (1.3)	2 (1.4)	>0.999
Known HF	34 (11.3)	17 (10.8)	17 (12.0)	0.741
Previous stroke/TIA	28 (9.3)	15 (9.5)	13 (9.2)	0.920
PAD	15 (5.0)	5 (3.2)	10 (7.0)	0.124
Anxiety	1 (0.3)	1 (0.6)	0 (0.0)	>0.999
Depression	4 (1.3)	2 (1.3)	2 (1.4)	>0.999
MCI/dementia	7 (2.3)	3 (1.9)	4 (2.8)	0.711
Chronic liver disease	14 (4.7)	7 (4.4)	7 (4.9)	0.838
Chronic kidney disease	34 (11.3)	15 (9.5)	19 (13.4)	0.289
Past COVID-19	11 (3.7)	6 (3.8)	5 (3.5)	0.899
HIV	1 (0.3)	1 (0.6)	0 (0.0)	>0.999
Labs at baseline, mean (SD)				
TG	1.8 (1.1)	1.7 (1.1)	1.8 (1.2)	0.478
TC	4.9 (1.4)	5.2 (1.3)	4.6 (1.5)	<0.001
LDL	3.1 (1.3)	3.4 (1.2)	2.8 (1.3)	<0.001
HDL	1.1 (0.3)	1.1 (0.3)	1.1 (0.3)	0.267
HbA1c	6.8 (1.8)	6.8 (1.7)	6.9 (1.9)	0.671
FPG	7.8 (3.7)	7.7 (3.8)	8.0 (3.5)	0.473
Medications at baseline, *n* (%)				
Aspirin	77 (25.7)	38 (24.1)	39 (27.5)	0.499
Any p2y12 inhibitors	29 (9.7)	13 (8.2)	16 (11.3)	0.374
OAC	6 (2.0)	2 (1.3)	4 (2.8)	0.427
BB	77 (25.7)	40 (25.3)	37 (26.1)	0.884
CCB	69 (23.0)	39 (24.7)	30 (21.1)	0.465
Nitrate	34 (11.3)	13 (8.2)	21 (14.8)	0.073
Ranolazine	0 (0.0)	0 (0.0)	0 (0.0)	N/A
Trimetazidine	4 (1.3)	2 (1.3)	2 (1.4)	>0.999
ACEi/ARB/ARNI	88 (29.3)	47 (29.7)	41 (28.9)	0.868
MRA	10 (3.3)	4 (2.5)	6 (4.2)	0.525
Any statins	140 (46.7)	74 (46.8)	66 (46.5)	0.951
Ezetimibe	25 (8.3)	11 (7.0)	14 (9.9)	0.365
Fibrates	10 (3.3)	6 (3.8)	4 (2.8)	0.753
PCSK9i	1 (0.3)	1 (0.6)	0 (0.0)	>0.999
Inclisiran	0 (0.0)	0 (0.0)	0 (0.0)	N/A
Other lipid lowering medications	0 (0.0)	0 (0.0)	0 (0.0)	N/A
All lipid lowering medications	145 (48.3)	77 (48.7)	68 (47.9)	0.883
Combination lipid lowering medications	29 (9.7)	14 (8.9)	15 (10.6)	0.618
SGLT2i	26 (8.7)	15 (9.5)	11 (7.7)	0.591
Other OHGAs	88 (29.3)	39 (24.7)	49 (34.5)	0.062
Insulin	24 (8.0)	9 (5.7)	15 (10.6)	0.121
Statin intensity at baseline, *n* (%)				0.761
Low intensity	14 (4.7)	8 (5.1)	6 (4.2)	
Moderate intensity	57 (19.0)	27 (17.1)	30 (21.1)	
High intensity	69 (23.0)	39 (24.7)	30 (21.1)	
Medications on discharge, *n* (%)				
Aspirin	287 (95.7)	153 (96.8)	134 (94.4)	0.294
Any p2y12 inhibitors	289 (96.3)	151 (95.6)	138 (97.2)	0.458
OAC	38 (12.7)	24 (15.2)	14 (9.9)	0.166
BB	253 (84.3)	133 (84.2)	120 (84.5)	0.937
CCB	32 (10.7)	19 (12.0)	13 (9.2)	0.421
Nitrate	157 (52.3)	81 (51.3)	76 (53.5)	0.696
Ranolazine	1 (0.3)	1 (0.6)	0 (0.0)	>0.999
Trimetazidine	6 (2.0)	5 (3.2)	1 (0.7)	0.218
ACEi/ARB/ARNI	220 (73.3)	121 (76.6)	99 (69.7)	0.179
MRA	23 (7.7)	13 (8.2)	10 (7.0)	0.700
Any statins	295 (98.3)	154 (97.5)	141 (99.3)	0.374
Ezetimibe	39 (13.0)	20 (12.7)	19 (13.4)	0.853
Fibrates	9 (3.0)	4 (2.5)	5 (3.5)	0.740
PCSK9i	0 (0.0)	0 (0.0)	0 (0.0)	N/A
Inclisiran	0 (0.0)	0 (0.0)	0 (0.0)	N/A
Other lipid lowering medications	1 (0.3)	1 (0.6)	0 (0.0)	>0.999
All lipid lowering medications	297 (99.0)	155 (98.1)	142 (100.0)	0.250
Combination lipid lowering medications	46 (15.3)	23 (14.6)	23 (16.2)	0.694
SGLT2i	35 (11.7)	18 (11.4)	17 (12.0)	0.876
Other OHGAs	115 (38.3)	56 (35.4)	59 (41.5)	0.277
Insulin	32 (10.7)	14 (8.9)	18 (12.7)	0.285
Statin intensity on discharge, *n* (%)				0.511
Low intensity	1 (0.3)	0 (0.0)	1 (0.7)	
Moderate intensity	7 (2.3)	4 (2.5)	3 (2.1)	
High intensity	287 (95.7)	150 (94.9)	137 (96.5)	
LVEF (%), mean (SD)	47.8 (11.8)	47.2 (11.2)	48.5 (12.4)	0.324
PCI prior to discharge, *n* (%)	235 (78.3)	122 (77.2)	113 (79.6)	0.620
CABG prior to discharge, *n* (%)	12 (4.0)	6 (3.8)	6 (4.2)	0.850
Repeat LDL	2.0 (0.9)	2.6 (0.9)	1.4 (0.3)	<0.001
Duration to repeat LDL (months), mean (SD)	9.5 (4.2)	9.9 (4.3)	9.0 (4.1)	0.081
Duration of follow-up (years), mean (SD)	1.0 (0.3)	0.9 (0.3)	1.0 (0.3)	0.621
Outcomes, *n* (%)				
All-cause mortality	12 (4.0)	5 (3.2)	7 (4.9)	0.436
CV mortality	10 (3.3)	4 (2.5)	6 (4.2)	0.525
ACS	29 (9.7)	13 (8.2)	16 (11.3)	0.374
Stroke/TIA	7 (2.3)	5 (3.2)	2 (1.4)	0.452
PAD	3 (1.0)	0 (0.0)	3 (2.1)	0.105
HF hospitalisation	32 (10.7)	19 (12.0)	13 (9.2)	0.421
MACE^a^	39 (13.0)	18 (11.4)	21 (14.8)	0.382
Number of readmissions per patient, mean (SD)	1.1 (0.4)	1.1 (0.4)	1.2 (0.5)	0.172
Average length of stay (days), mean (SD)	4.7 (5.2)	4.2 (5.9)	5.3 (4.3)	0.277
Number of outpatient clinic visits per patient, mean (SD)	3.2 (1.6)	3.2 (1.7)	3.0 (1.6)	0.278
Annualised cost ($), mean (SD)				
Statin medication	677 (1,108)	691 (1,167)	661 (1,042)	0.814
ED attendance + inpatient admission	144 (354)	139 (400)	151 (296)	0.764
Outpatient clinic visit	182 (170)	198 (213)	164 (100)	0.081
Subtotal medical service	1,003 (1,235)	1,028 (1,323)	976 (1,133)	0.715

a MACE included outcomes of CV mortality, ACS, stroke/TIA.

**Table 4:** Baseline characteristics, treatment patterns, and outcomes stratified by LDL-C target attainment at <1.4 mmol/L.

Variables	Overall *N* = 300	Did not reach target *N* = 245	LDL target <1.4 *N* = 55	*p*-value
Age (years), mean (SD)	60.9 (12.2)	61.1 (12.5)	59.8 (11.1)	0.471
Female, *n* (%)	54 (18.0)	49 (20.0)	5 (9.1)	0.057
Race, *n* (%)				0.061
Chinese	160 (53.3)	126 (51.4)	34 (61.8)	
Malay	58 (19.3)	54 (22.0)	4 (7.3)	
Indian	72 (24.0)	57 (23.3)	15 (27.3)	
Others	10 (3.3)	8 (3.3)	2 (3.6)	
Family history, *n* (%)				
IHD	14 (4.7)	11 (4.5)	3 (5.5)	0.726
Stroke	3 (1.0)	3 (1.2)	0 (0.0)	>0.999
Co-morbidities, *n* (%)				
Previous or current smoking	140 (46.7)	113 (46.1)	27 (49.1)	0.690
DM	129 (43.0)	100 (40.8)	29 (52.7)	0.107
HLD	210 (70.0)	176 (71.8)	34 (61.8)	0.143
FH	4 (1.3)	2 (0.8)	2 (3.6)	0.155
HTN	178 (59.3)	143 (58.4)	35 (63.6)	0.472
Prothrombotic state	16 (5.3)	13 (5.3)	3 (5.5)	>0.999
AF	26 (8.7)	21 (8.6)	5 (9.1)	>0.999
Known IHD (non-AMI)	24 (8.0)	19 (7.8)	5 (9.1)	0.783
Previous CIED implantation	4 (1.3)	3 (1.2)	1 (1.8)	0.557
Known HF	34 (11.3)	26 (10.6)	8 (14.5)	0.406
Previous stroke/TIA	28 (9.3)	23 (9.4)	5 (9.1)	0.945
PAD	15 (5.0)	13 (5.3)	2 (3.6)	>0.999
Anxiety	1 (0.3)	1 (0.4)	0 (0.0)	>0.999
Depression	4 (1.3)	3 (1.2)	1 (1.8)	0.557
MCI/dementia	7 (2.3)	7 (2.9)	0 (0.0)	0.357
Chronic liver disease	14 (4.7)	12 (4.9)	2 (3.6)	>0.999
Chronic kidney disease	34 (11.3)	25 (10.2)	9 (16.4)	0.193
Past COVID-19	11 (3.7)	9 (3.7)	2 (3.6)	>0.999
HIV	1 (0.3)	1 (0.4)	0 (0.0)	>0.999
Labs at baseline, mean (SD)				
TG	1.8 (1.1)	1.7 (1.1)	1.8 (1.3)	0.756
TC	4.9 (1.4)	5.1 (1.3)	4.3 (1.7)	<0.001
LDL	3.1 (1.3)	3.2 (1.2)	2.5 (1.5)	<0.001
HDL	1.1 (0.3)	1.1 (0.3)	1.0 (0.3)	0.224
HbA1c	6.8 (1.8)	6.8 (1.8)	7.0 (1.8)	0.392
FPG	7.8 (3.7)	7.7 (3.7)	8.3 (3.8)	0.397
Medications at baseline, *n* (%)				
Aspirin	77 (25.7)	60 (24.5)	17 (30.9)	0.325
Any p2y12 inhibitors	29 (9.7)	23 (9.4)	6 (10.9)	0.730
OAC	6 (2.0)	4 (1.6)	2 (3.6)	0.303
BB	77 (25.7)	63 (25.7)	14 (25.5)	0.968
CCB	69 (23.0)	57 (23.3)	12 (21.8)	0.818
Nitrate	34 (11.3)	26 (10.6)	8 (14.5)	0.406
Ranolazine	0 (0.0)	0 (0.0)	0 (0.0)	N/A
Trimetazidine	4 (1.3)	4 (1.6)	0 (0.0)	>0.999
ACEi/ARB/ARNI	88 (29.3)	69 (28.2)	19 (34.5)	0.347
MRA	10 (3.3)	8 (3.3)	2 (3.6)	>0.999
Any statins	140 (46.7)	115 (46.9)	25 (45.5)	0.842
Ezetimibe	25 (8.3)	18 (7.3)	7 (12.7)	0.186
Fibrates	10 (3.3)	8 (3.3)	2 (3.6)	>0.999
PCSK9i	1 (0.3)	1 (0.4)	0 (0.0)	>0.999
Inclisiran	0 (0.0)	0 (0.0)	0 (0.0)	N/A
Other lipid lowering medications	0 (0.0)	0 (0.0)	0 (0.0)	N/A
All lipid lowering medications	145 (48.3)	119 (48.6)	26 (47.3)	0.862
Combination lipid lowering medications	29 (9.7)	21 (8.6)	8 (14.5)	0.175
SGLT2i	26 (8.7)	17 (6.9)	9 (16.4)	0.034
Other OHGAs	88 (29.3)	67 (27.3)	21 (38.2)	0.111
Insulin	24 (8.0)	18 (7.3)	6 (10.9)	0.409
Statin intensity at baseline, *n* (%)				0.797
Low intensity	14 (4.7)	13 (5.3)	1 (1.8)	
Moderate intensity	57 (19.0)	47 (19.2)	10 (18.2)	
High intensity	69 (23.0)	55 (22.4)	14 (25.5)	
Medications on discharge, *n* (%)				
Aspirin	287 (95.7)	238 (97.1)	49 (89.1)	0.018
Any p2y12 inhibitors	289 (96.3)	234 (95.5)	55 (100.0)	0.226
OAC	38 (12.7)	31 (12.7)	7 (12.7)	0.988
BB	253 (84.3)	210 (85.7)	43 (78.2)	0.165
CCB	32 (10.7)	26 (10.6)	6 (10.9)	0.949
Nitrate	157 (52.3)	126 (51.4)	31 (56.4)	0.508
Ranolazine	1 (0.3)	1 (0.4)	0 (0.0)	>0.999
Trimetazidine	6 (2.0)	6 (2.4)	0 (0.0)	0.597
ACEi/ARB/ARNI	220 (73.3)	182 (74.3)	38 (69.1)	0.431
MRA	23 (7.7)	19 (7.8)	4 (7.3)	>0.999
Any statins	295 (98.3)	240 (98.0)	55 (100.0)	0.589
Ezetimibe	39 (13.0)	31 (12.7)	8 (14.5)	0.706
Fibrates	9 (3.0)	7 (2.9)	2 (3.6)	0.672
PCSK9i	0 (0.0)	0 (0.0)	0 (0.0)	N/A
Inclisiran	0 (0.0)	0 (0.0)	0 (0.0)	N/A
Other lipid lowering medications	1 (0.3)	1 (0.4)	0 (0.0)	>0.999
All lipid lowering medications	297 (99.0)	242 (98.8)	55 (100.0)	>0.999
Combination lipid lowering medications	46 (15.3)	36 (14.7)	10 (18.2)	0.516
SGLT2i	35 (11.7)	25 (10.2)	10 (18.2)	0.096
Other OHGAs	115 (38.3)	88 (35.9)	27 (49.1)	0.069
Insulin	32 (10.7)	26 (10.6)	6 (10.9)	0.949
Statin intensity on discharge, *n* (%)				0.515
Low intensity	1 (0.3)	1 (0.4)	0 (0.0)	
Moderate intensity	7 (2.3)	7 (2.9)	0 (0.0)	
High intensity	287 (95.7)	232 (94.7)	55 (100.0)	
LVEF (%), mean (SD)	47.8 (11.8)	47.7 (11.7)	48.2 (12.4)	0.775
PCI prior to discharge, *n* (%)	235 (78.3)	190 (77.6)	45 (81.8)	0.488
CABG prior to discharge, *n* (%)	12 (4.0)	11 (4.5)	1 (1.8)	0.702
Repeat LDL	2.0 (0.9)	2.2 (0.9)	1.1 (0.2)	<0.001
Duration to repeat LDL (months), mean (SD)	9.5 (4.2)	9.6 (4.2)	8.9 (3.9)	0.237
Duration of follow-up (years), mean (SD)	1.0 (0.3)	0.9 (0.3)	1.0 (0.3)	0.411
Outcomes, *n* (%)				
All-cause mortality	12 (4.0)	11 (4.5)	1 (1.8)	0.702
CV mortality	10 (3.3)	9 (3.7)	1 (1.8)	0.695
ACS	29 (9.7)	22 (9.0)	7 (12.7)	0.395
Stroke/TIA	7 (2.3)	5 (2.0)	2 (3.6)	0.616
PAD	3 (1.0)	1 (0.4)	2 (3.6)	0.088
HF hospitalisation	32 (10.7)	27 (11.0)	5 (9.1)	0.675
MACE^a^	39 (13.0)	30 (12.2)	9 (16.4)	0.412
Number of readmissions per patient, mean (SD)	1.1 (0.4)	1.1 (0.4)	1.1 (0.4)	0.918
Average length of stay (days), mean (SD)	4.7 (5.2)	4.4 (5.5)	6.2 (3.4)	0.153
Number of outpatient clinic visits per patient, mean (SD)	3.2 (1.6)	3.1 (1.6)	3.2 (2.0)	0.732
Annualised cost ($), mean (SD)				
Statin medication	677 (1,108)	661 (1,049)	750 (1,349)	0.589
ED attendance + inpatient admission	144 (354)	142 (371)	155 (266)	0.799
Outpatient clinic visit	182 (170)	185 (181)	168 (111)	0.503
Subtotal medical service	1,003 (1,235)	987 (1,193)	1,073 (1,420)	0.641

a MACE included outcomes of CV mortality, ACS, stroke/TIA.

In univariable comparisons, patients who achieved LDL-C <2.6 mmol/L were older than those who did not reach target and differed by ethnicity and smoking status. Baseline total cholesterol, LDL-C, and HbA1c were also significantly different between groups. Across the <1.8 mmol/L and <1.4 mmol/L thresholds, lower baseline total cholesterol and LDL-C were consistently associated with subsequent goal attainment. No consistent differences were observed in discharge statin intensity, ezetimibe use, revascularisation status, or LVEF across LDL-C target groups.

The median time to repeat LDL-C testing was 9.9 months (IQR 6.6–12.7), with a mean of 9.5 ± 4.2 months. At one year (±1 month), 80.7% (*n* = 242) achieved the local Ministry of Health goal of <2.6 mmol/L, 47.3% (*n* = 142) achieved <1.8 mmol/L, and only 18.3% (*n* = 55) reached the very high-risk target of <1.4 mmol/L. Intermediate control rates were observed in 29.0% (*n* = 87) for LDL-C between 1.4 and <1.8 mmol/L, and in 33.3% (*n* = 100) for LDL-C between 1.8 and <2.6 mmol/L (Table 5).

**Table 5:** LDL-C goal attainment and distribution of LDL-C categories at one-year follow-up.

LDL-C category	Threshold (mmol/L)	*n* (%)
ESC/EAS very-high-risk target	<1.4	55 (18.3%)
Intermediate threshold	<1.8	142 (47.3%)
Intermediate control	1.4 to <1.8	87 (29.0%)
MOH goal	<2.6	242 (80.7%)
Intermediate control	1.8 to <2.6	100 (33.3%)

LDL-C, low-density lipoprotein cholesterol; ESC/EAS, European Society of Cardiology/European Atherosclerosis Society; MOH, Ministry of Health. Threshold-based goal attainment categories are not mutually exclusive: patients achieving <1.4 mmol/L are also included within the <1.8 mmol/L and <2.6 mmol/L groups. Intermediate LDL-C categories are mutually exclusive.

At baseline, 48.3% (*n* = 145) of patients were prescribed LLT, with 9.7% (*n* = 29) receiving combination therapy. Statins were used in 46.7% (*n* = 140), ezetimibe in 8.3% (*n* = 25), fibrates in 3.3% (*n* = 10), and PCSK9 inhibitors in 0.3% (*n* = 1). At discharge, 99.0% (*n* = 297) were on LLT, with combination therapy in 15.3% (*n* = 46). Statin prescription increased to 98.3% (*n* = 295), ezetimibe to 13.0% (*n* = 39), and fibrates remained stable at 3.0% (*n* = 9). No patients were discharged on PCSK9 inhibitors or inclisiran. High-intensity statin use rose from 23.0% (*n* = 69) at baseline to 95.7% (*n* = 287) at discharge (consistent with local policy recommendations for high-intensity statin therapy after MI), while those not on statins decreased from 53.3% (*n* = 160) to 1.7% (*n* = 5).

During the 12-month follow-up period, 9.7% (*n* = 29) developed new acute coronary syndrome, 2.3% (*n* = 7) had a stroke or transient ischaemic attack, and 1.0% (*n* = 3) developed peripheral artery disease. Subsequent ASCVD events occurred in 12.7% (*n* = 38) of patients, with only one patient (0.3%) experiencing a second subsequent event. Recurrent events of the same ASCVD type as the index event occurred in 9.7% (*n* = 29).

LDL-C control was not significantly associated with major adverse cardiovascular events (MACE) at 12 months. For LDL- ≥2.6 mmol/L vs. <2.6 mmol/L, MACE occurred in 10.3% vs. 13.6% (*p* = 0.503); for LDL-C ≥1.8 mmol/L vs. <1.8 mmol/L, MACE rates were 11.4% vs. 14.8% (*p* = 0.382); and for LDL-C ≥1.4 mmol/L vs. <1.4 mmol/L, MACE rates were 12.2% vs. 16.4% (*p* = 0.412). No significant differences were observed in all-cause mortality, cardiovascular mortality, acute coronary syndrome, stroke/TIA, peripheral artery disease, or heart failure hospitalisation across LDL-C categories.

The burden of ASCVD-related healthcare utilisation over a mean follow-up of 1.0 ± 0.3 years was moderate. Patients had a mean of 1.1 ± 0.4 emergency department visits or hospital readmissions, a mean hospital stay of 4.7 ± 5.2 days, and 3.2 ± 1.6 outpatient visits. The mean annualised cost of statin therapy SGD 677 ± 1,108, ED attendance and inpatient admission SGD 144 ± 354, and outpatient clinic visits SGD 182 ± 170, giving an estimated combined annualised cost of SGD 1,003 per patient.

## Discussion

In this real-world cohort of 300 post-myocardial infarction patients in Singapore, LDL-C goal attainment at one year was strikingly suboptimal despite near-universal use of high-intensity statins. The mean patient age was 60.9 years, and the cohort exhibited a high burden of cardiometabolic comorbidities, with hyperlipidaemia, hypertension, and diabetes present in 70.0%, 59.3%, and 43.0% of patients, respectively. At one year, 80.7% achieved the local Ministry of Health threshold of <2.6 mmol/L, only 47.3% attained the intermediate threshold of <1.8 mmol/L, and merely 18.3% reached the ESC/EAS very-high-risk target of <1.4 mmol/L. Combination lipid-lowering therapy remained infrequently prescribed—ezetimibe was used in just 13.0% of patients and PCSK9 inhibitors were almost absent—despite high rates of statin prescription (98.3%) at discharge. Mean time to repeat lipid testing was prolonged at 9.5 months, indicating delayed monitoring and therapeutic adjustment. Over a mean follow-up of one year, 13.0% of patients experienced MACE, with no statistically significant difference in event rates across LDL-C categories. The absence of a significant association between LDL-C target attainment and 12-month MACE should be interpreted cautiously and should not be taken to contradict the established benefit of LDL-C lowering. This finding likely reflects the modest sample size, limited number of MACE events, short follow-up duration, residual confounding, and lack of time-updated LDL-C, adherence, or follow-up treatment data. Accordingly, the outcome analysis should be considered exploratory, with the main contribution of this study being the characterisation of real-world LDL-C goal attainment and treatment gaps in post-MI care. The mean annualised direct healthcare cost per patient, while not inclusive of procedures and cardiac rehabilitation costs, was SGD 1,003. This reflects a moderate but measurable economic burden, given that the annual myocardial infarction case burden in Singapore exceeds 11,000 cases per year (20). Collectively, these findings underscore persistent therapeutic gaps in LDL-C goal attainment and the potential missed opportunity for early combination therapy and systematic follow-up in post-MI secondary prevention.

The apparent disconnect between near-universal high-intensity statin prescription at discharge and poor attainment of stricter LDL-C targets is clinically important. Several mechanisms may explain this gap. First, the expected LDL-C reduction from high-intensity statin monotherapy may be insufficient for patients presenting with high baseline LDL-C, particularly when the therapeutic goal is <1.4 mmol/L. Second, delayed repeat lipid testing, observed at a median of approximately 10 months in this cohort, limits opportunities for early treatment intensification during the period of highest recurrent risk after MI. Third, real-world adherence may decline after discharge; local evidence suggests that up to one-third of Singaporean patients fail to adhere consistently to chronic cardiovascular medications, including statins (21). International studies report even higher figures, with nearly 50% of patients discontinuing therapy within the first year (22). Patient-reported reasons for poor adherence are diverse, ranging from a preference for lifestyle modification and general aversion to medications to pill burden and fear of adverse effects (23). Genetic predispositions, such as SLCO1B1 polymorphisms, further contribute to statin intolerance in Asian populations, amplifying reluctance to persist with therapy (24). Finally, therapeutic inertia and restricted access to non-statin agents may delay escalation to ezetimibe, PCSK9 inhibitors, or inclisiran. These factors suggest that suboptimal LDL-C control is unlikely to reflect statin under-prescription alone, but rather a combination of biological, behavioural, and health-system barriers. In our cohort, lower baseline LDL-C and total cholesterol appeared to be the most consistent factors associated with subsequent LDL-C goal attainment. This suggests that patients presenting with higher baseline LDL-C may be less likely to reach stringent targets with statin-based therapy alone, supporting early identification of patients unlikely to achieve <1.4 mmol/L and consideration of upfront or early add-on ezetimibe.

To contextualise our findings, Table 6 compares LDL-C goal attainment and lipid-lowering treatment patterns in the present cohort with prior local, regional, and international real-world studies. Our findings are consistent with earlier Singaporean data, where fewer than half of ischaemic heart disease patients achieved <1.8 mmol/L and only 22.1% reached <1.4 mmol/L at two years (25). Similarly, the A-SACT (Achievement in Singapore of Cholesterol Targets) study reported that up to 94% of very high-risk patients in Singapore failed to meet <70 mg/dL (∼1.8 mmol/L) goals almost two decades ago (26). These persistent gaps align with findings from regional registries such as DYSIS II, which demonstrated LDL-C attainment rates of only 31% in stable coronary disease and 23% in ACS across the Asia-Pacific region (15). Contemporary European real-world evidence from the SANTORINI study similarly demonstrated incomplete LDL-C goal attainment, although combination lipid-lowering therapy uptake was higher than in the present cohort (27). Taken together, these data highlight a persistent evidence–practice gap across real-world settings, particularly in the uptake of combination lipid-lowering therapy. This gap persists despite robust trial evidence—including FOURIER and ODYSSEY—demonstrating that more intensive LDL-C lowering with PCSK9 inhibitors reduces recurrent cardiovascular events in high-risk populations (28, 29).

**Table 6:** Comparison of LDL-C goal attainment and lipid-lowering treatment patterns across real-world studies.

Study	Country/region	Population	Follow-up	LDL-C target assessed	LDL-C goal attainment	Lipid-lowering treatment pattern
Present study	Singapore	300 consecutive post-MI patients	12 months ± 1 month	<2.6, <1.8, and <1.4 mmol/L	80.7% achieved <2.6 mmol/L; 47.3% achieved <1.8 mmol/L; 18.3% achieved <1.4 mmol/L	High-intensity statin use increased to 95.7% at discharge; ezetimibe use remained low at 13.0%; no patients were discharged on PCSK9 inhibitors or inclisiran
Mak et al., (25)	Singapore	Ischaemic heart disease patients	2 years	<1.8 and <1.4 mmol/L	Fewer than half achieved <1.8 mmol/L; 22.1% achieved <1.4 mmol/L	Not primarily focused on post-MI discharge prescribing
A-SACT, 2006	Singapore	Very-high-risk patients with established atherothrombotic disease	Cross-sectional/registry-based	<70 mg/dL (∼<1.8 mmol/L)	Up to 94% of very-high-risk patients failed to achieve LDL-C <70 mg/dL	Demonstrated substantial LDL-C undertreatment in very-high-risk Singapore patients
DYSIS II Singapore, 2019	Singapore	325 patients: 199 stable CHD and 126 ACS	Baseline; ACS followed to 4 months	<70 mg/dL (∼<1.8 mmol/L)	At baseline, LDL-C <70 mg/dL was achieved by 28.1% of treated CHD patients and 20.2% of treated ACS patients; among initially untreated ACS patients, attainment improved from 0% to 54.5% at 4 months.	LLT use was common but not optimised; treated patients had lower LDL-C than untreated patients
DYSIS II Asia-Pacific	Asia-Pacific	Stable CHD and ACS patients	Baseline; ACS followed post-discharge	<70 mg/dL (∼<1.8 mmol/L)	LDL-C attainment approximately 31% in stable coronary disease and 23% in ACS	Treatment intensification remained inadequate in many patients
SANTORINI, 2024	14 European countries	9,602 high- or very-high cardiovascular risk patients (complete baseline data)	Baseline (published); 1-year follow-up published in 2024; data presented at EAS 2023	2019 ESC/EAS risk-based goals: <1.8 mmol/L for high-risk and <1.4 mmol/L for very high-risk	Baseline goal attainment: 20.3% overall. At 1-year follow-up (conference data): approximately one-third achieved risk-based LDL-C goals	Combination therapy at baseline: 24.0%. At 1-year follow-up (conference data): increased from 25.6% to 37.9% overall
SWEDEHEART, 2025	Sweden	35,826 LLT-naive MI patients discharged on statins	Median 3.96 years	Timing of ezetimibe initiation after MI	Not primarily a goal-attainment study; evaluated outcomes by early, late, or no ezetimibe use. 1-year MACE incidence: 1.79 (early), 2.58 (late), 4.03 (no ezetimibe) per 100 patient-years	16.9% received ezetimibe early (≤12 weeks), 18.1% late, and 65.0% no ezetimibe; high-intensity statin use ≥98% in all groups

LDL-C, low-density lipoprotein cholesterol; MI, myocardial infarction; LLT, lipid-lowering therapy; CHD, coronary heart disease; ACS, acute coronary syndrome; ESC/EAS, European Society of Cardiology/European Atherosclerosis Society. Comparisons across studies are descriptive only, as study populations, follow-up duration, LDL-C thresholds, and reported treatment patterns differed between cohorts.

Furthermore, our study demonstrated that the use of combination lipid-lowering therapy (LLT) was infrequent and suboptimal, increasing only modestly from 9.7% at baseline to 15.3% at discharge. A prior Singapore study similarly reported low uptake of combination therapy, although its rates were higher, rising from 12.3% at baseline to 33.8% by the end of follow-up (15). However, the figures are not directly comparable, as the prior study has a longer follow-up period, reflecting greater opportunity for therapy intensification. Nevertheless, both studies highlight a consistent pattern of underutilisation of combination therapy in secondary prevention. The persistently low adoption likely reflects a combination of clinical, system-level, and socioeconomic barriers. Clinicians may continue to favour a stepwise approach—maximising statin monotherapy before adding adjunct agents—while cost and reimbursement constraints remain major deterrents, as ezetimibe, PCSK9 inhibitors, and inclisiran were not universally subsidised in Singapore's public healthcare system at the time of study. Limited clinician familiarity with newer agents, coupled with the absence of structured follow-up pathways for early LDL-C reassessment, may further delay escalation. Together, these barriers may explain the low use of combination therapy observed in this cohort. Evidence from both registries and randomised trials strongly supports earlier intensification. In SWEDEHEART, post-MI patients initiated on ezetimibe within 12 weeks were more likely to achieve LDL-C goals and experienced fewer recurrent cardiovascular events (30). The PL-ACS registry likewise demonstrated lower three-year mortality with statin–ezetimibe combination therapy compared with statin monotherapy (5.5% vs. 10.2%) (31). Randomised trials reinforce these observations: IMPROVE-IT confirmed that adding ezetimibe to statins reduced LDL-C by approximately 15 mg/dL and lowered MACE risk by 6% (32); ODYSSEY OUTCOMES showed that alirocumab reduced MACE by −15% post-ACS (29); and FOURIER demonstrated a 59% LDL-C reduction and significant outcome improvement with evolocumab in stable ASCVD (28). More recently, the VICTORION-INCEPTION trial reported that inclisiran enabled −67% of ACS patients to achieve LDL-C <70 mg/dL at one year vs. −28% under usual care (33). Fixed-dose combination therapy may represent another practical strategy to improve adherence and simplify treatment intensification. Single-pill combinations of statins with ezetimibe may reduce pill burden, improve persistence, and facilitate earlier combination therapy in patients unlikely to achieve LDL-C targets with statin monotherapy. However, availability, regulatory approval, patent restrictions, formulary inclusion, and subsidy status may limit their use in some healthcare systems, including Singapore. Future implementation strategies should therefore consider not only access to advanced agents such as PCSK9 inhibitors and inclisiran, but also lower-cost adherence-enhancing approaches such as fixed-dose statin–ezetimibe combinations where available (34, 35). Collectively, these findings support earlier and more intensive LDL-C lowering, and provide a strong rationale for proactive combination therapy in very-high-risk patients.

In practical terms, our results point towards a structured post-MI lipid optimisation pathway rather than a sequential, statin-only escalation model. Patients with high baseline LDL-C or those unlikely to achieve target with statin monotherapy should be considered for early ezetimibe initiation before or soon after discharge. Repeat lipid testing should be scheduled within 4–12 weeks, rather than deferred to routine long-term follow-up, to allow timely intensification. For patients who remain above target despite maximally tolerated statin and ezetimibe therapy, early referral for PCSK9 inhibitor or inclisiran consideration may be appropriate, particularly in very high-risk patients. Embedding these steps into discharge checklists, pharmacist-led lipid clinics, or cardiology follow-up protocols may reduce therapeutic inertia and improve secondary prevention.

From a health economics perspective, the mean direct cost of SGD 1,003 per patient likely underestimates the true burden, as it excludes indirect costs such as productivity loss, long-term disability, and premature mortality, as well as cardiac procedures and cardiac rehabilitation. Cost-effectiveness analyses provide supportive evidence for early intensification. The IMPROVE-IT economic evaluation estimated an incremental cost-effectiveness ratio (ICER) of USD 40,000 per quality-adjusted life-year (QALY) gained (36). Similarly, analyses of FOURIER demonstrated that evolocumab was cost-effective in selected very-high-risk subgroups once the high costs of recurrent events were considered (37). Although region-specific analyses are needed for Asia, these data suggest that early adoption of combination therapy in high-risk groups could yield both clinical and economic benefits.

This study has several limitations. First, its retrospective observational design introduces a risk of unmeasured and residual confounding, as socioeconomic factors, lifestyle behaviours, medication adherence, and treatment persistence were not systematically captured. Second, this was a single-centre study conducted at a tertiary cardiac centre in Singapore, which may limit generalisability to other institutions with different patient demographics, referral patterns, prescribing practices, and financing models. Third, although consecutive sampling was used, the sample size was modest and the number of MACE events was limited, reducing statistical power to detect differences in clinical outcomes across LDL-C categories or to perform robust subgroup analyses. Fourth, follow-up was limited to approximately one year, which may not have been sufficient to capture the full cardiovascular benefit of LDL-C reduction. This may partly explain the absence of a significant association between LDL-C attainment and clinical outcomes. Fifth, follow-up LDL-C testing and lipid-lowering therapy titration were not standardised, and follow-up LLT prescription, persistence, and adherence data were not systematically captured. Therefore, changes in statin intensity, ezetimibe initiation, treatment discontinuation, or escalation to advanced agents during follow-up could not be fully assessed. Finally, the cost analysis was restricted to selected direct medical costs and excluded indirect societal costs and costs of cardiac procedures such as coronary artery bypass grafting and percutaneous coronary intervention. Nevertheless, the use of consecutive patient sampling, standardised data collection, and adjudicated endpoint definitions supports the internal validity of the findings.

Despite the limitations, these findings carry important policy implications for Singapore. At the health-system level, these findings support broader access to non-statin lipid-lowering therapies, particularly for very high-risk post-MI patients who remain above target despite maximally tolerated statin therapy. Expanded subsidy schemes for ezetimibe, PCSK9 inhibitors, and inclisiran, together with national LDL-C audit programmes and structured patient engagement strategies, may improve accountability, adherence, and real-world implementation of guideline-directed lipid management.

Future research should prioritise multicentre registries with longer follow-up, incorporating adherence and socioeconomic data to provide a more comprehensive picture of LDL-C control in Singapore. Pragmatic real-world trials of upfront combination therapy at discharge are particularly warranted to determine whether aggressive strategies can meaningfully improve long-term outcomes in Asian populations.

## Conclusion

In this real-world cohort of post-MI patients in Singapore, LDL-C target attainment remained suboptimal, particularly at the more stringent thresholds recommended for very high-risk patients, despite near-universal prescription of high-intensity statins at discharge. No significant association between LDL-C control and 1-year MACE was observed, which may reflect the limited follow-up duration, modest number of clinical events, and residual confounding. These findings suggest that earlier consideration of combination lipid-lowering therapy, structured follow-up pathways, and adherence-focused strategies may help narrow the gap between guideline recommendations and clinical practice.

Beyond clinical outcomes, suboptimal LDL-C control in this high-risk population may be associated with greater healthcare resource utilisation and economic burden through recurrent cardiovascular events, repeat hospitalisations, and long-term secondary prevention costs. Further multicentre studies with longer follow-up are needed to determine whether coordinated improvements in clinical practice, patient engagement, and healthcare policy can improve LDL-C target attainment and reduce residual cardiovascular risk in high-risk Asian populations.

## Data Availability

The original contributions presented in the study are included in the article/Supplementary Material, further inquiries can be directed to the corresponding author/s.
