# Supplementary material for: Real-world assessment of low-density lipoprotein cholesterol goal, disease burden and financial burden among patients with atherosclerotic cardiovascular disease in Singapore—A single-centre retrospective cohort study
Source: Front Cardiovasc Med. 2026 Jun 12;13:1815535. doi: 10.3389/fcvm.2026.1815535 (PMC13303499; doi:10.3389/fcvm.2026.1815535)
Supplement: Supplementary file 1 [file Table1.docx]

**Supplementary Table 1.** Sample size power calculation

| Power Analysis of One Proportion  Numeric Results for testing H0: P = P0 versus H1: P < P0  Test Statistic: Exact Test | | | | | | | |
| --- | --- | --- | --- | --- | --- | --- | --- |
| Power | N | Proportion Given H0 (P0) | Proportion Given H1 (P1) | Target Alpha | Actual Alpha | Beta | Reject H0 If R<=Value |
| 0.9003 | 75 | 0.3500 | 0.2000 | 0.0500 | 0.0485 | 0.0997 | 19 |
| 0.8032 | 55 | 0.3500 | 0.2000 | 0.0500 | 0.0489 | 0.1968 | 13 |
| 0.9064 | 108 | 0.3500 | 0.2200 | 0.0500 | 0.0449 | 0.0936 | 29 |
| 0.8008 | 75 | 0.3500 | 0.2200 | 0.0500 | 0.0485 | 0.1992 | 19 |
| 0.9003 | 149 | 0.3500 | 0.2400 | 0.0500 | 0.0470 | 0.0997 | 42 |
| 0.8060 | 111 | 0.3500 | 0.2400 | 0.0500 | 0.0462 | 0.1940 | 30 |
| 0.9032 | 230 | 0.3500 | 0.2600 | 0.0500 | 0.0472 | 0.0968 | 68 |
| 0.8028 | 168 | 0.3500 | 0.2600 | 0.0500 | 0.0462 | 0.1972 | 48 |
| 0.9014 | 386 | 0.3500 | 0.2800 | 0.0500 | 0.0470 | 0.0986 | 119 |
| 0.8033 | 279 | 0.3500 | 0.2800 | 0.0500 | 0.0482 | 0.1967 | 84 |
| 0.9004 | 764 | 0.3500 | 0.3000 | 0.0500 | 0.0476 | 0.0996 | 245 |
| 0.8004 | 545 | 0.3500 | 0.3000 | 0.0500 | 0.0498 | 0.1996 | 172 |
| 0.9001 | 2140 | 0.3500 | 0.3200 | 0.0500 | 0.0486 | 0.0999 | 712 |
| 0.8002 | 1544 | 0.3500 | 0.3200 | 0.0500 | 0.0491 | 0.1998 | 509 |
| 0.9003 | 19379 | 0.3500 | 0.3400 | 0.0500 | 0.0500 | 0.0997 | 6673 |
| 0.8003 | 13995 | 0.3500 | 0.3400 | 0.0500 | 0.0500 | 0.1997 | 4805 |
